# CT radiomics nomogram predicts pathological response after induced chemotherapy and overall survival in patients with advanced laryngeal cancer: A single-center retrospective study

**DOI:** 10.3389/fonc.2023.1094768

**Published:** 2023-03-24

**Authors:** Chunmiao Kang, Pengfeng Sun, Runqin Yang, Changming Zhang, Wenfeng Ning, Hongsheng Liu

**Affiliations:** ^1^ Department of Ultrasound, Shaanxi Provincial People’s Hospital, Xi’an, China; ^2^ Department of Radiology, Xi’an Central Hospital Affiliated to Xi’an Jiaotong University, Xi’an, Shaanxi, China; ^3^ Department of Otolaryngology, Xijing Hospital, Air Force Military Medical University, Xi’an, China

**Keywords:** laryngeal, chemotherapy, pathological response, radiomics, nomogram

## Abstract

**Purpose:**

This study aimed to develop a radiomics nomogram to predict pathological response (PR) after induction chemotherapy (IC) and overall survival (OS) in patients with advanced laryngeal cancer (LC).

**Methods:**

This retrospective study included patients with LC (n = 114) who had undergone contrast computerized tomography (CT); patients were randomly assigned to training (n = 81) and validation cohorts (n = 33). Potential radiomics scores were calculated to establish a model for predicting the PR status using least absolute shrinkage and selection operator (LASSO) regression. Multivariable logistic regression analyses were performed to select significant variables for predicting PR status. Kaplan–Meier analysis was performed to assess the risk stratification ability of PR and radiomics score (rad-score) for predicting OS. A prognostic nomogram was developed by integrating radiomics features and clinicopathological characteristics using multivariate Cox regression. All LC patients were stratified as low- and high-risk by the median CT radiomic score, C-index, calibration curve. Additionally, decision curve analysis (DCA) of the nomogram was performed to test model performance and clinical usefulness.

**Results:**

Overall, PR rates were 45.6% (37/81) and 39.3% (13/33) in the training and validation cohorts, respectively. Eight features were optimally selected to build a rad-score model, which was significantly associated with PR and OS. The median OS in the PR group was significantly shorter than that in the non-PR group in both cohorts. Multivariate Cox analysis revealed that volume [hazard ratio, (HR) = 1.43], N stage (HR = 1.46), and rad-score (HR = 2.65) were independent risk factors associated with OS. The above four variables were applied to develop a nomogram for predicting OS, and the DCAs indicated that the predictive performance of the nomogram was better than that of the clinical model.

**Conclusion:**

For patients with advanced LC, CT radiomics score was an independent biomarker for estimating PR after IC. Moreover, the nomogram that incorporated radiomics features and clinicopathological factors performed better for individualized OS estimation.

## Introduction

Among the respiratory systems, laryngeal cancer (LC) is the leading cause of cancer-related deaths and the third most common cancer of the head and neck (1). The treatment principle of laryngeal cancer relies on the premise of complete tumor removal and maximal retention or reconstruction of laryngeal function to improve the quality of life of patients (2, 3). Although total laryngectomy (TL) remains the gold standard for advanced LC, induction chemotherapy (IC) with or without radiotherapy as a standard approach for larynx preservation commonly recommends addressing advanced LC before TL in the current NCCN guidelines (4); it achieves better larynx preservation than immediate surgery without considering overall survival (OS) (5, 6). Toya et al. (7) reported that IC provides excellent pathological response (PR) with acceptable toxicities in T3N0 glottic carcinoma without vocal cord fixation (7); however, there is a risk of toxic accumulation during IC in patients with poor PR. Thus, it is important to predict the PR with high precision to guide appropriate treatment strategies.

Response evaluation criteria in solid tumors (RECIST) are currently used to evaluate the efficacy of IC (8). However, the uncertain growth morphology of laryngeal carcinoma and insignificant hemodynamic changes caused by IC limits the application of CT. In addition, traditional enhanced CT is less sensitive in predicting therapeutic response, especially PR after IC therapy. Although removal of the contrasted CT-defined area of the LC is considered standard care after IC, approximately 40% of patients may still have to undergo TL even after IC (9). Thus, the early identification of patients who respond poorly to IC is crucial before TL. Compared to conventional CT, radiomics is an emerging method for imaging analysis using algorithms or statistical analysis tools to capture distinct phenotypic differences of tumors from diagnostic images (10, 11). Radiomics has shown its powerful potential in the diagnosis and prognosis prediction of some tumors beyond the morphological characteristics of traditional CT (12). To the best of our knowledge, no study has introduced a radiomics analysis to predict PR after IC therapy in patients with advanced LC. We assumed that radiomic features would have an advantage over traditional CT for predicting PR after IC in patients with locally advanced LC to select patients who require immediate TL.

## Materials and methods

### Patients

**Figure 1:**
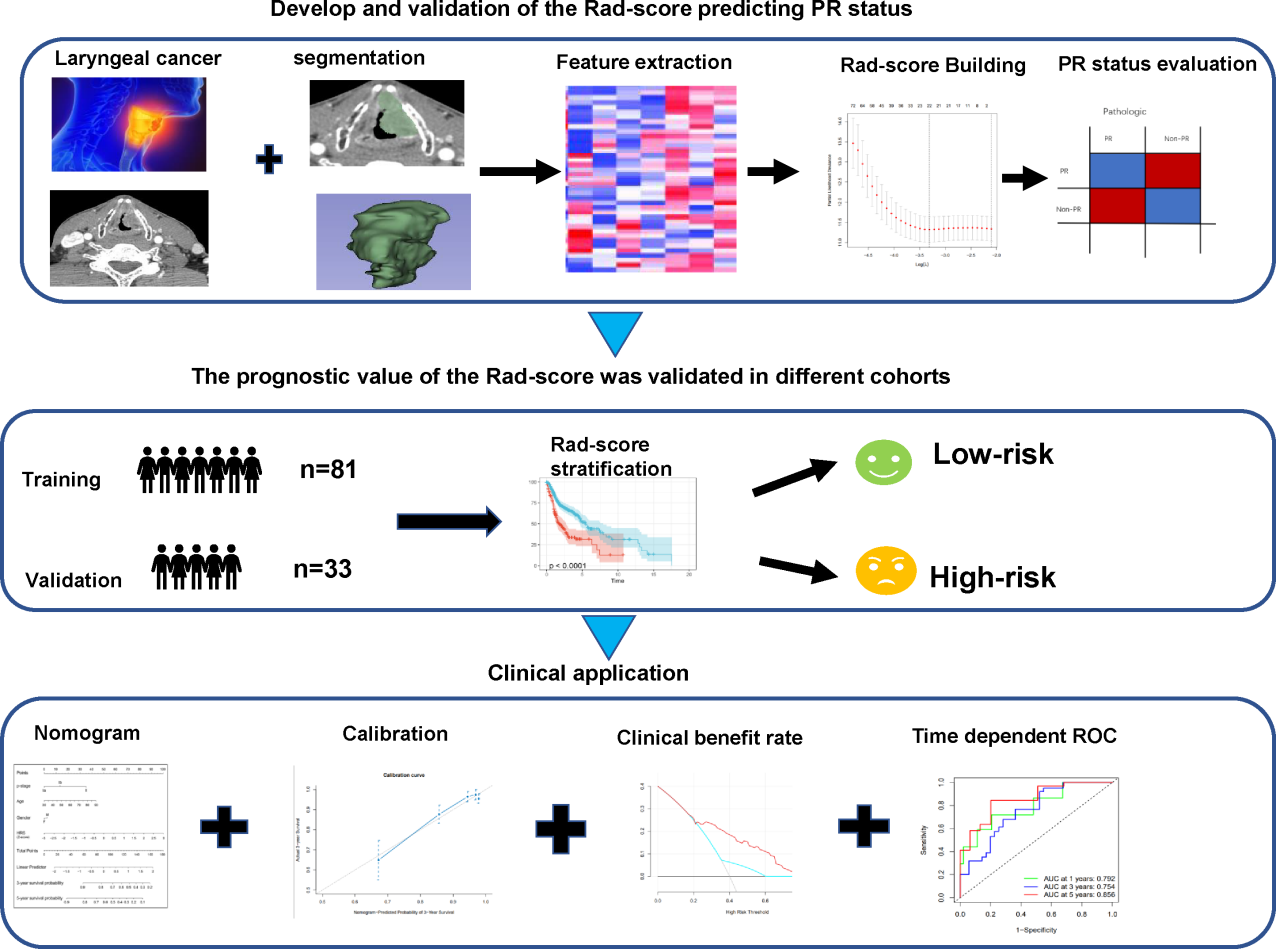
Workflow of CT radiomic score model development for predicting pathological response status and OS in advanced LC patients. CT, computed tomography; OS, overall survival; LC, laryngeal cancer.

A total of 114 patients diagnosed with LC from Xi’an Central Hospital were enrolled from June 1, 2013, to July 31, 2021, and a technical roadmap was designed for this study (**Figure 1**). The study protocol was approved by the Medical Ethics Review Committee of the Shaanxi Provincial People’s Hospital, and the need for informed consent was waived. The development history of the Shaanxi Provincial People’s Hospital is interconnected with the development and fate of the country and nation, which was located in Xi’an, Shaanxi Province, Northwest China. It is a large comprehensive three-level Grade public hospital directly under the provincial government, integrating medical care, emergency aid, teaching, scientific research, cadre health care and rehabilitation. The hospital was characterized by cardiovascular medicine, medical imaging and otolaryngology, and was also one of the three medical centers in the Northwest. The hospital covers an area of 106,000 square meters and a building area of 315,000 square meters. It has 10 medical centers, 75 clinical medical and technical departments, and 21 national key specialties, provincial key disciplines and provincial advantageous medical specialties. Tumors were staged according to the American Joint Committee on Cancer (AJCC) staging system. The inclusion criteria were as follows (1): primary LC confirmed pathologically and *via* biopsy, pre-IC clinical newly diagnosed with resectable cT_3–4_N_0–3_ stage, (2) CT performed prior to IC therapy before 1 week with artifact-free imaging data, and (3) availability of clinicopathologic and follow-up data. The exclusion criteria were as follows: (1) patients with multiple cancers; (2) minimum diameter of the tumor <1 cm with at least 2 slices on CT, insufficient to contain a region of interest (ROI); (3) occurrence of an adverse event during IC therapy or less than 3 cycles of IC; (4) >7 weeks duration between CT scan and initial IC therapy. Primary endpoint was pathological response after IC therapy in patients with advanced LC. The secondary endpoint of all patients was OS, calculated from the day of diagnosis to the date of death from the disease. Local recurrence and distant metastasis were diagnosed based on clinical symptoms, physical examination, and imaging findings (including MRI, ultrasound, contrast-enhanced CT, and whole-body bone scans). A total of 114 consecutive patients who met the criteria were enrolled and divided into two cohorts (training and validation cohorts) at a ratio of 7:3 using computer-generated random numbers, which was consistent with previous studies (13, 14).

Treatment and Endpoint

All eligible LC patients administered three cycles of TPF protocols (docetaxel on day 1, cisplatin on day 1, and fluorouracil 750 per day) at 3-week intervals. Premedication consisted of oral dexamethasone (twice a day) and ciprofloxacin (500 mg twice a day). In case of febrile neutropenia, granulocyte colony-stimulating factor was given. All patients received laryngoscopy or neck CT scan after the third treatment cycle to evaluate the treatment response. Before treatment, all patients provided written informed consent. At baseline, they underwent clinical assessments including endoscopy and neck CT; primary endpoint (pathological response) assessed by pathologist after three cycles IC followed surgery.

### Defining pathological response

All patients underwent biopsy and surgical resection within two weeks of CT examination before IC therapy. All tissues were reviewed by a pathologist with more than 10 years of experience in head and neck pathology to estimate the TN stage of the tumor. The status of pathological T for each LC tumor was determined by the surgical pathological results within 1 month after IC. Patients were classified in

response categories as proposed by Vos et al (15, 16): Complete response was defined as no invasive tumor in the larynx and no lymph nodes (pT0/Tis N0); partial response was defined as a small size (≤50% residual viable tumor cell percentage in the resected tumor bed) remaining in the lesion, with vocal cord activity not having been fixed. Responding patients (pathological response, PR) were classified as having either complete or partial response. Patients with any percentage of residual viable tumor cells but < 50% regression had no pathological response (Non-PR), including no obvious change or larger compared with the pre-IC tumor.

### Laryngeal CT imaging

Laryngeal CT images were acquired rapidly during a single breath-hold in the craniocaudal direction. All patients were scanned using a Philips Brilliance-iCT256 scanner. The scanning parameters were as follows: tube voltage, 120 kV; tube current, 250mA; field of view (FOV), 500 mm; matrix, 512 × 512 mm; and scan thickness, 0.9 mm. For the enhancement scan, 60–80 mL of non-ionic contrast agent was injected through the elbow vein using a high-pressure syringe (iodixanol, 320 mg/ml, Byer, 1.5 mL/kg and 2 mL/s). A free-hand, phase-based, individualized ROI outlining the whole tumor profile was manually and independently drawn on contrasted images by two radiologists, and the CT value was automatically generated. In this study, the metastatic lymph nodes were not included in ROI.

### Image processing and region of interest (ROI) segmentation

3D-Slicer software was used to isotropically resample the CT images in the arterial and venous phases with a voxel size of 1×1×1 mm. The ROI segmentation was delineated around the tumor outline for the largest cross-sectional area in the axial CT image. ROI segmentation should avoid the cartilage, tissue containing air contents, and pre-epiglottic and paraglottic spaces. To analyze the intra- and inter-class correlation coefficients, 40 patients were randomly selected to repeatedly redraw the ROI 1 month after the first delineation to improve the reliability and reproducibility of the radiomics features.

### Radiomics feature extraction

3D-slicer was used to automatically extract the radiomic features. In total, 851 radiomics features were extracted separately from the delineated ROI in the arterial and venous phases. Eight types of features were extracted from the contrast CT images: first-order (n = 32), gray-level dependence matrix (GLDM, n = 14), gray-level co-occurrence matrix (GLCM, n = 24), gray-level run length matrix (GLRLM, n = 16), gray-level size zone matrix (GLSZM, n = 16), neighboring gray tone difference matrix (NGTDM, n = 5), and wavelet-based features (n = 744). All texture features are summarized and defined in detail in **Supplementary Table 1**. The features were extracted by discretizing the CT values of the ROI based on a fixed interval width (bin width = 25 HU).

### Feature selection and radiomics signature construction

Intra/interclass correlation coefficients (ICCs) greater than 0.8 were considered as high stability features. To avoid overfitting, the least absolute shrinkage and selection operator (LASSO) method with 100 iterations of 10-fold cross-validation was used to screen the optimal significant features from the training cohort. The radiomics score (rad-score) was calculated as follows: (Rad-score = $\sum^{}\text{feature }1\;\times \text{ corresponding coefficient }1\;+\text{ feature }2\;\times \text{ corresponding coefficient }2\ldots$ )..

### Development of PR and OS prediction models

All variables (including conventional radiological, radiomic, and baseline clinical characteristics of patients) were evaluated for predicting PR status using univariable and multivariable logistic regression analyses. Univariate and multivariate Cox regression analyses were used to determine independent prognostic factors of OS. All patients were subsequently stratified into high- and low-risk groups according to the median rad-score. A prognostic nomogram was developed by integrating radiomics features and clinicopathological characteristics using multivariate Cox regression. The calibration curves were plotted using Hosmer-Lemeshow tests to evaluate the calibration of the prognostic nomogram. The Harrell’s C-index was calculated to quantify the diagnostic power of the nomogram. Decision curve analysis (DCA) was performed to calculate the net clinical benefits in the training and validation cohorts.

### Follow-up and statistical analysis

All statistical tests were performed using the R statistical software (version 4.0.2). OS was measured from the first day after LC diagnosis until death. Survival analysis was performed using the “survminer” package. “Rms” and “pec” packages were utilized to build nomogram and calibration plots *via* R software. DCA curves were generated using the “rmda” and “ggDCA” packages in R. DeLong’s test (*p* < 0.05) was used to compare the performance (C-index and AUCs) of various models.

## Results

A total of 159 patients diagnosed with locally advanced LC treated with IC therapy were enrolled in the study, of which 11 patients received chemotherapy (less than three cycles); five patients (4%) did not complete the follow-up. Of the 5 LC patients that follow up was not completed, 3 reported with wrong information and phone number, 2 LC patients had transportation barriers. The median follow-up time for those still alive at the last follow-up was 25 (range: 15.67–63.03 months). Follow-up data showed that 28% of deaths (32/114) occurred during the follow-up period: 20 due to cancer and 12 due to other diseases. The median time between CT and surgery was 20 days (range: 4–79 days). Only 114 patients who met the inclusion criteria were suitable for extracting texture features for the study. As a result, 81 patients were allocated to the training cohort, and 33 patients were allocated to the independent validation cohort. **Supplementary Figure S1** shows a flowchart depicting the number of patients included in the analysis after exclusion criteria. PR to IC therapy was detected in 45.6% (37/81) of LC cases in the training cohort, similar to the 39.4% (13/33) seen in the validation cohort. Clinicopathological features of the patients with advanced LC enrolled in this study are shown in **Table 1**.** **A flowchart of the study is presented in **Figure 1**.

**Table 1:** The clinical and imaging characteristics of primary cohort.

Variables	Training cohort (n=81)		Validation cohort (33)	
	PR N=37	Non-PR N=44	PR N=13	Non-PR N=20
**Age**	46.7 ± 10.2	47.8 ± 11.3	43.7 ± 9.2	42.8 ± 7.3
Gender				
Female	7	10	4	5
male	30	34	9	15
Site				
Supraglottic	16	11	6	9
glottic	21	33	7	11
T Stage				
T3	15	20	8	11
T4	20	24	5	9
N Stage				
N0	27	13	6	13
N1-3	10	31	7	7
Radiation				
Yes	24	25	9	8
No	13	19	4	12
**Size(cm)**	11.1 ± 0.41	10.3 ± 0.54	10.2 ± 0.73	9.4 ± 0.52
**Volume(cm^3^)**	15.28 ± 1.01	11.27 ± 0.07	13.25 ± 0.56	10.26 ± 0.27

### Radiomic feature extraction, selection, and model building

Spearman’s correlation coefficients (ICC > 0.8) were calculated to obtain the most highly stable features (n = 275), which were selected for univariate Cox and LASSO Cox regression analyses (**Supplementary Figure S2**). As shown in **Supplementary Table 2**, these features (n = 6) were identified as independent prognostic factors for development of the rad-score model. The corresponding coefficients of the eligible features were calculated to build the rad-score predicting PR in the training sets. The rad-score formula was as follows: GLSZM -SizeZoneNonUniformity×1.21 + GLCM-Cluster Prominence×1.23-GLDM-Large Dependence Emphasis×3.21 + LHH-gldm-Large Dependence Emphasis×0.08 + HHL-GLDM-Large Dependence Emphasis×3.74 + NGTDM-Complexity×0.48. We further revealed whether the rad-score was associated with Clinicopathological information. Our study presented that the no significant correlation between radiomic score with T, N, tumor site and gender except PR status (**Supplementary Figure S3**).

### Development of predictive models for PR status

**Figure 2:**
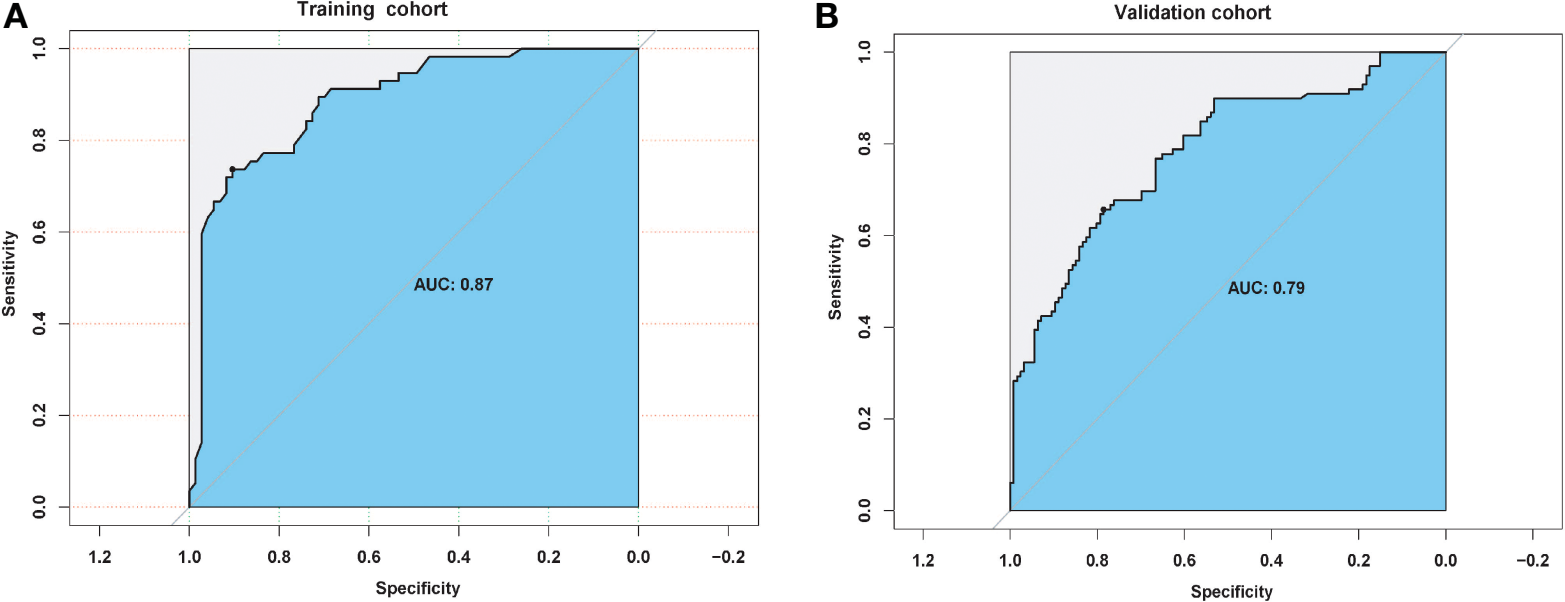
Predictive pathological response status performance of radiomic score in **(A)** training and **(B)** validation cohorts.

**Figure 3:**
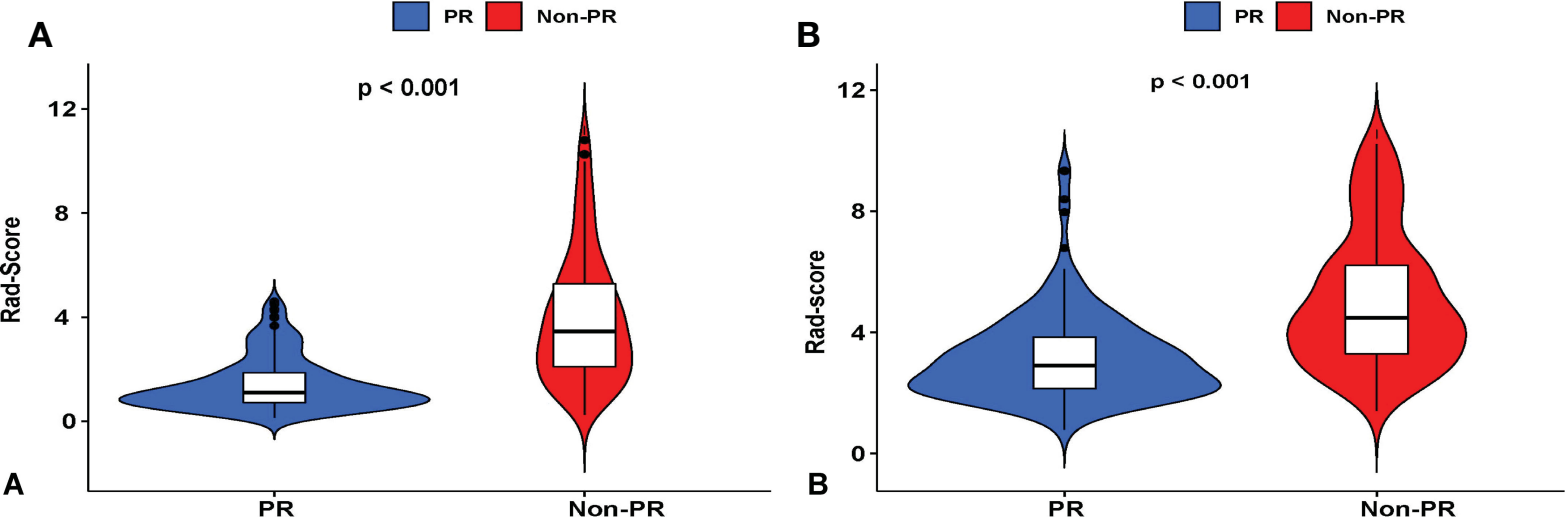
Violin plots showing that the radiomic score (rad-score) of the non-pathological response (PR) group is substantially higher than that of the PR of LC patients who underwent IC therapy in **(A)** training and **(B)** validation cohorts. Red represents a high rad-score in the non-PR group, and blue represents a low rad-score in the PR group.

Univariate and multivariate logistic analyses were used to determine the relationship between clinicopathological features, quantitative radiological parameters, and PR status. From the results of univariate analyses, a significant association was observed between rad-score and PR status in the training cohort; however, quantitative radiological parameters and tumor size were not related to PR status (**Table 2**). Multivariate logistic analysis demonstrated that only the rad-score was an independent predictor of the PR status (**Table 3**). None of the clinical factors were found to be independent predictors of PR status in the training and validation sets (**Supplementary Table 3**). The ROC curves of the rad-score for predicting PR in the training and validation cohorts are shown in **Figure 2**. In addition, rad-score distributions were estimated in the PR and non-PR groups, and patients had higher rad-scores in the PR group than in the non-PR group in the training and validation sets (**Figure 3**).

**Table 2:** Logistics regression analyses for PR status in the training cohort.

Variables	B	SE	Wald	Significance	
				univariate	multivariable
Age	0.37	0.29	1.61	0.20	
Female	-0.31	0.30	1.13	0.28	
Site	0.12	0.65	0.03	0.85	
T stage	0.36	0.28	1.43	0.32	
N Stage	0.97	0.67	2.11	0.15	
Radiation	-0.31	0.37	0.007	0.93	
Size	0.32	0.79	0.16	0.69	
Volume	0.67	0.35	3.70	0.06	
Rad-score	2.16	0.67	10.70	<0.001	<0.001

**Table 3:** Univariate and multivariate analysis with Cox proportional hazard model in Training cohort.

Covariates	Univariate		multivariate	
	HR(95%CI)	*P* value	HR(95%CI)	Adjusted *P* value
**Rad-score**	2.56(1.47-4.42)	0.001	2.65(1.42-4.95)	0.008
**N Stage**	1.32(1.09-3.19)	0.008	1.46(1.41-2.09)	0.04
**Volume**	1.44(1.25-2.34)	0.02	1.43(1.23-3.67)	0.04
**T Stage**	1.78(0.68-2.45)	0.32		
**Size**	1.35(0.68-3.78)	0.48		
**Radiation**	0.73(0.59-1.08)	0.57		
**Age**	1.36(0.89-2.34)	0.13		
**Female**	0.82(0.51-1.34)	0.44		
**Site**	0.73(0.41-1.35)	0.32		

Multivariate analysis was applied using the Cox proportional hazards(P<0.05) model to identify independent predictors of survival that involved the univariate variables. After a series of multivariate analyses, covariates with a P value < 0.05 were used for subsequent model construction. HR, hazard ratios.

### Radiomics nomograms building for predicting OS`

**Figure 4:**
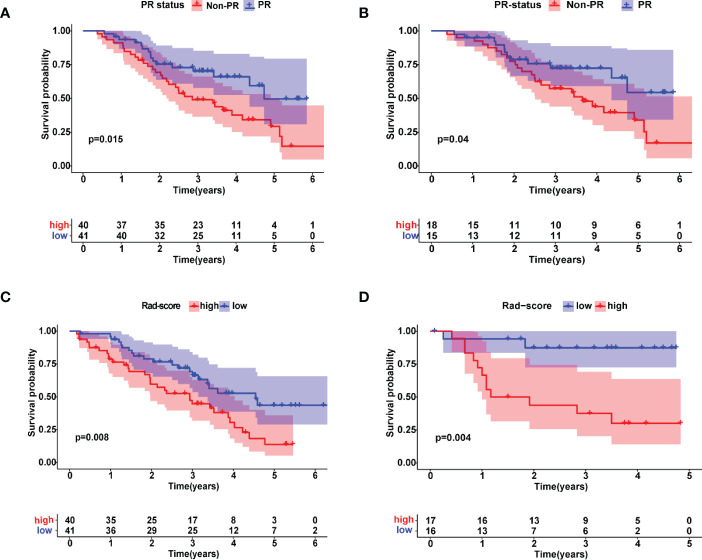
Kaplan–Meier estimates of overall survival with the associated 95% confidence intervals. Patients were stratified by pathological response (PR) status in **(A)** training and **(B)** validation cohorts. Blue and red lines represent patients in PR and non-PR groups, respectively. Patients were stratified by radiomic scores in **(C)** training and **(D)** validation cohorts. Blue and red lines represent patients in lower and higher risk groups, respectively.

**Figure 5:**
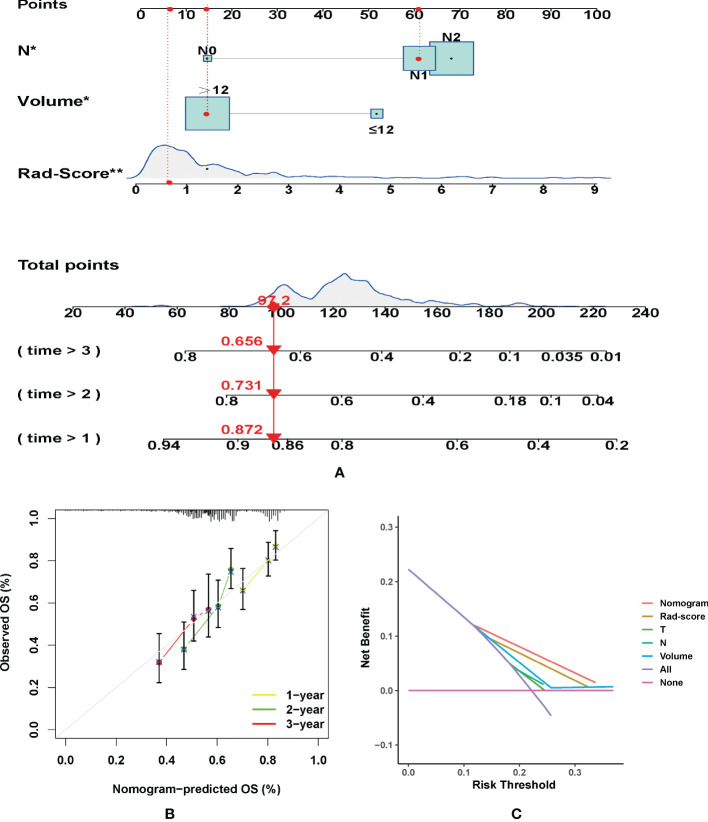
**(A)** Nomogram for predicting the overall survival (OS) of advanced laryngeal cancer (LC) patients. For each patient, lines are drawn downward to assess the points received from the four prognostic factors in the nomogram. The sum of these points is shown on the “Total points” axis. A line is drawn downward to determine the 1-, 2-, and 3-year OS of LC patients. **(B)** Calibration plot for the internal validation of the nomogram. The Y-axis exhibits actual survival. The X-axis exhibits nomogram-estimated survival. The dotted line (45° diagonal line) signifies full agreement between actual and observed probabilities. **(C)** Clinical net benefit of the nomogram, radiomic score, and other clinical features (including T, N, and Volume). *P =0.04, **P =0.008.

According to the Kaplan-Meier analysis, patients with PR had greater OS than patients without PR (*p* = 0.015, **Figure 4A**) in the training cohort. Similar results were also observed in the validation cohort (**Figure 4B**). The survival status and radiomic score distributions suggested that patients with higher rad-scores had worse outcomes (**Figures 4C, D**) in the training and validation cohorts. The relationship between OS, clinical factors, and imaging biomarkers was explored using R software. Univariate Cox analysis revealed that two clinical variables (including T and N), tumor volume, and rad-score were identified as significant biomarkers. Further multivariate Cox analysis showed that the N status, and rad-score were independent predictors of OS in both cohorts (**Table 3** and **Supplementary Table 4**). These above variables were applied to develop the nomogram (**Figure 5A**) for predicting OS, yielding a 1-year C-index of 0.802 in the training cohort and 0.735 in the validation cohort. The ROC curves for the different prediction models are presented in **Table 4**. The calibration curves for predicting the probability of OS at 1, 2, and 3 years after 1000 bootstrap simulations are shown in **Figure 5B**. The calibration curve results suggest that the observed probability agreed with the actual probability. DCAs showed that the nomogram had a higher overall net benefit than the clinical and pathological models (**Figure 5C**).

**Table 4:** Compare with clinical and radiomic model predicting OS of LC patients.

AUC	Cohorts	Clinical Model		Radiomic Model		CR Model
		T	N	Volume	Rad-score	Nomogram
1-year	Training set	0.591	0.635	0.673	0.747	0.802
	Validation set	0.543	0.561	0.604	0.704	0.735
3-year	Training set	0.563	0.642	0.667	0.737	0.789
	Validation set	0.534	0.587	0.643	0.702	0.746

## Discussion

In the current study, we developed and validated a radiomics feature-based nomogram and a clinical nomogram for predicting the PR status and OS of patients with advanced LC. More specifically, our initial results revealed that the rad-score was an independent predictor of PR status after adjusting for all clinical variables. Meanwhile, our data also showed that the contrasted-CT-derived rad-score and PR status successfully stratified patients into high- and low-risk groups. Importantly, the low-risk group tended to have lower rad-scores with favorable OS in the training and validation cohorts. The nomogram integrating rad-score and clinical factors performed better than the clinical model alone, yielding a C-index of 0.8 in the training cohort and 0.73 in the validation cohort. Pre-operatively predicting PR who undergo IC therapy and OS in patients with advanced LC is important for those in high-risk groups to avoid excessive toxicity accumulation replaced with surgery.

In this study, the PR-based radiomics model was established based on eight radiomics features, including features obtained from the venous phase, which were more than those obtained from the arterial phase. Chen et al. (18) developed CT-based models derived from venous phase to predict pathological complete response after neoadjuvant chemotherapy for advanced gastric cancer, yielding satisfactory accuracy with a C-index of 0.76 (18). Venous phase features provide more comprehensive information on tumor heterogeneity than AP features. Among all eligible features, the rad-score was developed based on two first-order features and six textural features. First-order features reflect the distribution of voxel intensities within tumors. The skewness and kurtosis of CT reflect the asymmetry of pixel intensity distribution and sharpness of the pixel intensity distribution, respectively (19). Cheng et al. (20) extracted textural features from PET images and predicted the prognosis for 88 patients with T3 or T4 oropharyngeal cancer, confirming that higher values of zone-size non-uniformity were identified as a worse predictor of progression-free survival and disease-specific survival (20). Similarly, the GLSZM Size Zone Non-Uniformity in our study was used to build a rad-score to predict the PR status and OS in patients with advanced LC. Cluster prominence showed significant differences between responders and partial responders to chemotherapy in a study predicting response to chemotherapy in a cohort of breast cancer patients (21, 22). In addition, the small area emphasis derived from pre-treatment ADC images has the potential to predict tumor resistance to neoadjuvant chemoradiotherapy in locally advanced rectal cancer (23). GLCM features have been reported to be sensitive to hypoxia and are significantly associated with survival in lung cancer and renal clear cell carcinoma (24). Wang et al. (25) demonstrated that GLCM features correlate with FGFR1 status in head and neck cancers (25). An analysis of the correlation between GLSZM features (rad-score) and ADRB1 expression revealed that ADRB1 expression was significantly expressed in patients with low rad-scores (26). These feature characteristics might indicate that the texture features of the lesion may suggest the possibility of PR status with a higher value characterization of tumor pathology. Among all the clinical characteristics analyzed, T, N, and tumor size had limited predictive value for IC therapy efficacy. The latest research developed a radiomics model to predict long-term local control and laryngectomy-free survival in locally advanced squamous cell carcinoma of the laryngopharynx, and the results indicated that only texture was statistically significant with laryngectomy-free survival after adjusting for all the clinical variables (T stage, N stage) (19). Our results are consistent with these results; however, we included comprehensive risk factors (tumor volume) and tumor sites in our study. Notably, satisfactory results were observed in the internal verification cohorts.

Multivariate Cox analysis revealed that volume, N stage, and rad-score were statistically significant predictors of OS. Compared to PR patients, non-PR patients had significantly worse OS (*p* = 0.01), which is consistent with previous studies showing that non-PR patients had a poor prognosis for LC (27). The prognostic significance also differed between the PR and non-PR groups in both cohorts, consistent with the results of previous studies (28, 29). In the current study, we also observed that a larger volume (12 cm^3^) was a high-risk factor for OS. To date, an optimal cut-off has not yet been reported. However, the T stage was not statistically significant in the multivariable Cox analysis of survival prognosis. Rather than the tumor volume, the tumor T stage for LC is defined as the invasion scope of the disease (30). For the N stage, while undergoing neck dissection did not significantly affect survival, patients with nodal metastasis had a shorter survival than patients with advanced laryngeal cancer (31). Patients with lymph node metastasis included in this study were of the supraglottic type, and neck dissection was performed following surgery. Generally, the prognosis of the supraglottic type is worse than that of the glottic type, and the N stage is an independent risk factor. The small sample size makes it difficult to further stratify the subgroups to explore the relationship between site and outcomes. A previous study proved that radiomic features are an independent prognostic biomarker for LC (32), which agrees with our results.

The nomogram revealed that tumor heterogeneity was more useful than TN stage in predicting the OS of LC patients who underwent IC therapy following surgery. A radiomic model was developed in this study using traditional contrast CT images. The C-index of our model (0.802) was higher than that of pre-treated CT-based radiomic studies predicting PR status after IC therapy by 0.62 and 0.69 (17, 33). This suggests that our nomogram model may feature more tumor heterogeneity information than those studies. The low-risk group, based on the definition of radiomic score, is more likely to achieve pathological remission. Additionally, PR patients tend to have a good prognosis. Previous studies suggested that LC patients with larger tumors may be better served by upfront surgery than definitive IC therapy (29, 34). Although there was no significant difference in tumor volume between PR and non-PR patients, there was a significant difference in OS. The above findings demonstrate that tumor volume can effectively reflect tumor invasiveness compared to T stage, and multivariate Cox regression also confirmed this result. Otolaryngology clinicians can use the nomogram to select high-risk patients and select a suitable treatment plan for individual patients.

Although this novel nomogram demonstrated favorable accuracy, several limitations should be noted. Immune checkpoint inhibitors therapy has become the standard of care for platinum-refractory recurrent/metastatic head and neck squamous cell carcinoma (HNSCC), the majority of patients present chemotherapy resistance and do not benefit from targeted therapy. Taking into consideration the potentially severe immune-related toxicities and their high cost, 5 recurrent/metastatic LC patients undergoing IC were enrolled and evaluable (after IC ≥24 weeks) in this study. The small sample size is uncontrollable and insufficient for subgroup analysis, future large sample and multi-center research will help solve this problem. Besides, it was performed in a single hospital with a small sample size and lacked external validation. Multicenter prospective validation with a larger sample size is warranted to acquire more powerful evidence. Second, the clinical applications of radiomic algorithms and statistical analysis are relatively unfamiliar and complex. Last, not all patients’ therapy-related toxicities, complications, and voice quality were collected for subgroup analysis. Furthermore, gene transcriptome information was not considered in our study.

## Conclusion

This study provides a novel nomogram that incorporates both the radiomics signature and clinical risk factors for predicting the PR status and OS in patients with LC. Incorporating radiomics into a nomogram along with other clinicopathological risk factors to estimate the OS for advanced LC patients was more accurate than the clinical model alone. Our predictive models have potential, are non-invasive, and effectively complements the clinical practice.

## Data availability statement

The raw data supporting the conclusions of this article will be made available by the authors, without undue reservation.

## Ethics statement

The studies involving human participants were reviewed and approved by 2022CR1003KY. The patients/participants provided their written informed consent to participate in this study. Written informed consent was obtained from the individual(s) for the publication of any potentially identifiable images or data included in this article.

## Author contributions

Conceptualization: HL, Data curation: CZ and PS, Formal analysis: RY, Methodology: WN, Project Resources: CK, Software: PS, Supervision: CK, Visualization: HL. All authors contributed to the article and approved the submitted version.
