# Supplementary material for: CT radiomics nomogram predicts pathological response after induced chemotherapy and overall survival in patients with advanced laryngeal cancer: A single-center retrospective study
Source: Front Oncol. 2023 Mar 24;13:1094768. doi: 10.3389/fonc.2023.1094768 (PMC10103838; doi:10.3389/fonc.2023.1094768)
Supplement: Supplementary file 1 [file DataSheet_1.docx]

**SUPPLEMENTARY MATERIAL**

**
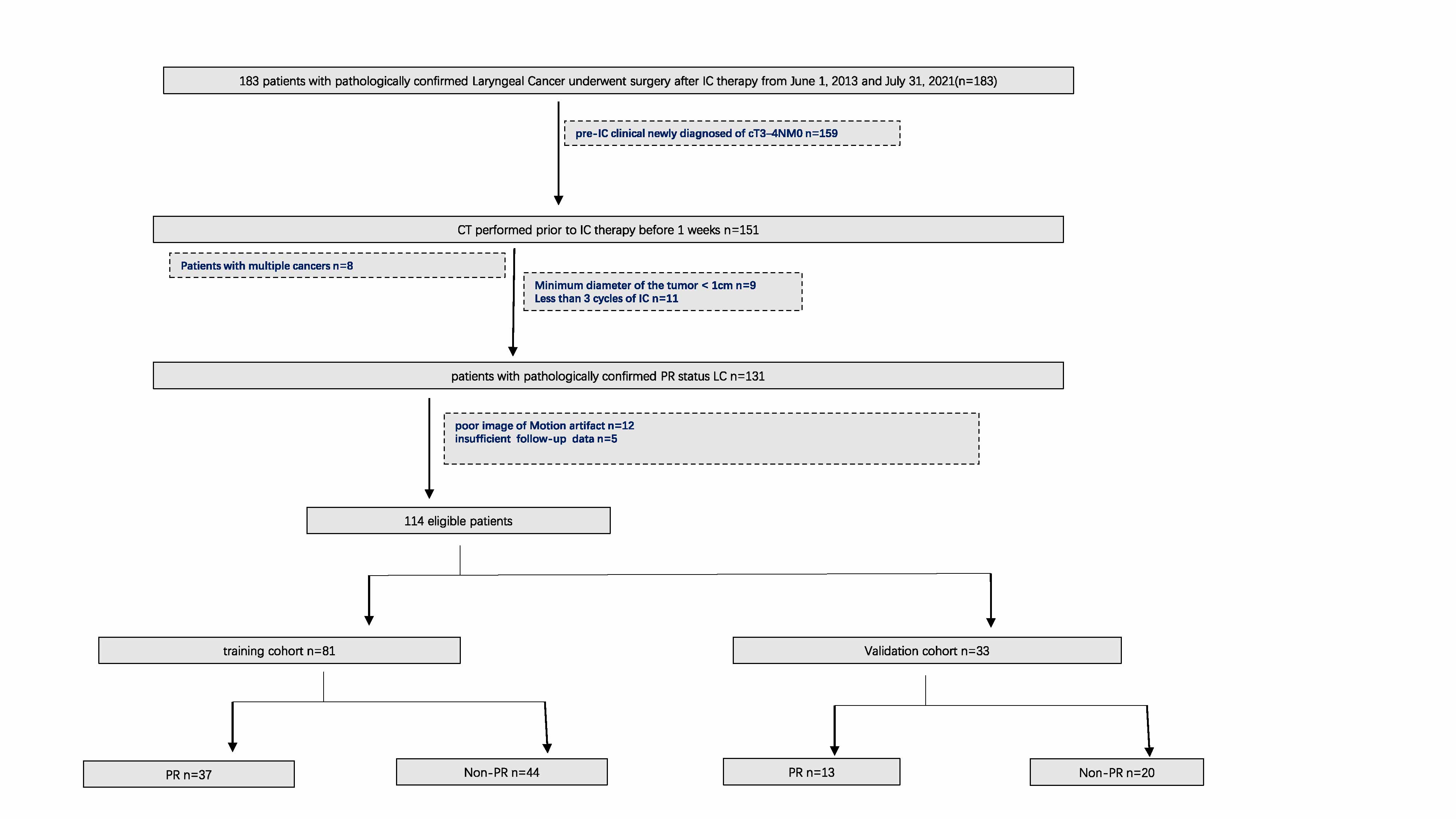
**

**Figure S1. Flowchart of enrolled LC patients in this study. CT, computed tomography. LC, Laryngeal carcinoma. IC, induced chemotherapy. PR, pathological response.**

**
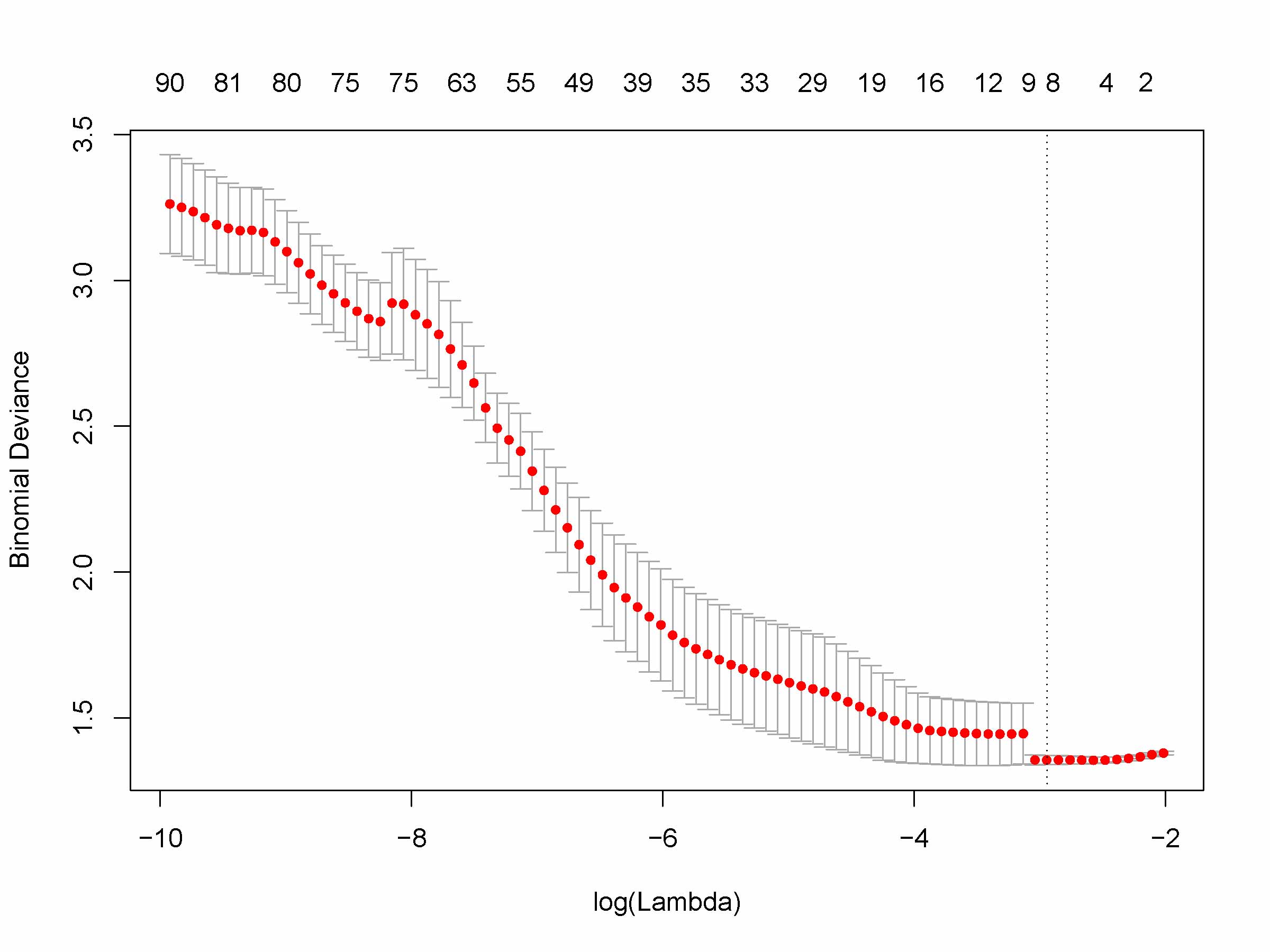
**

**Figure S2. Feature selection using the least absolute shrinkage and selection operator (LASSO) with a Cox regression model. The LASSO coefficient profile graph was plotted by coefficients against the log(lambda) sequence. Vertical lines were plotted for corresponding the 1-se and maximum criteria. As a result, 8 radiomic features with nonzero coefficients were selected (Supplementary Table2).**

**Supplementary Table**

**Table 1 The detailed information for all features**

| Features | | Numbers |
| --- | --- | --- |
| Intensity histogram |  | 18 |
| Texture | GLDM | 14 |
|  | GLCM | 24 |
|  | GLRLM | 16 |
|  | GLSZM | 16 |
|  | NGTDM | 5 |
| Shape |  | 14 |
| Wavelet | Wavelet-HLL | 93 |
|  | Wavelet-LHL | 93 |
|  | Wavelet-LHH | 93 |
|  | Wavelet-LLH | 93 |
|  | Wavelet-HLH | 93 |
|  | Wavelet-HHH | 93 |
|  | Wavelet-HHL | 93 |
|  | Wavelet-LLL | 93 |

**Supplementary Table 2**

**Table 2**. Radiomic signature building for predicting PR status in patients with advanced LC after IC therapy.

| **Feature** | **Phase** | **Coefficient** | **ICC value** |
| --- | --- | --- | --- |
| GLSZM Size Zone NonUniformity Normalized | VP | -1.21 | 0.92 |
| GLCM-Cluster Prominence | VP | 0.04 | 0.91 |
| LHH-GLSZM Small Area Emphasis | AP | -0.91 | 0.97 |
| HHH-GLRLM Low Gray Level Zone Emphasis | AP | 2.63 | 0.93 |
| HLH- GLCM- SumSquares | AP | -0.02 | 0.95 |
| HHL-GLSZM- Large Area Emphasis | AP | 0.32 | 0.92 |
| First-order- Skewness | AP | 0.53 | 0.97 |
| First-order- Kurtosis | AP | 1.43 | 0.98 |

**Supplementary Table 3**

**Table 3 Logistics regression analyses for PR status in the validation cohort.**

| **Variables** | **B** | **SE** | **Wald** | **Significance** | |
| --- | --- | --- | --- | --- | --- |
|  |  |  |  | **univariate** | **multivariable** |
| Age | 0.43 | 0.36 | 1.73 | 0.26 |  |
| Gender | -0.27 | 0.34 | 1.32 | 0.38 |  |
| Smoke | 0.54 | 0.45 | 3.12 | 0.08 |  |
| Location | 0.32 | 0.65 | 0.04 | 0.75 |  |
| T stage | 0.46 | 0.33 | 1.23 | 0.29 |  |
| N Stage | 0.88 | 0.77 | 2.31 | 0.18 |  |
| Radiation | -0.41 | 0.45 | 0.02 | 0.83 |  |
| Size | 0.42 | 0.69 | 0.25 | 0.64 |  |
| Volume | 0.56 | 0.43 | 3.45 | 0.08 |  |
| Enhancement Subtype | 0.73 | 0.45 | 9.88 | 0.09 |  |
| Rad-score | 2.14 | 0.57 | 10.54 | ＜0.001 | ＜0.001 |

**Supplementary FigureS3**

**
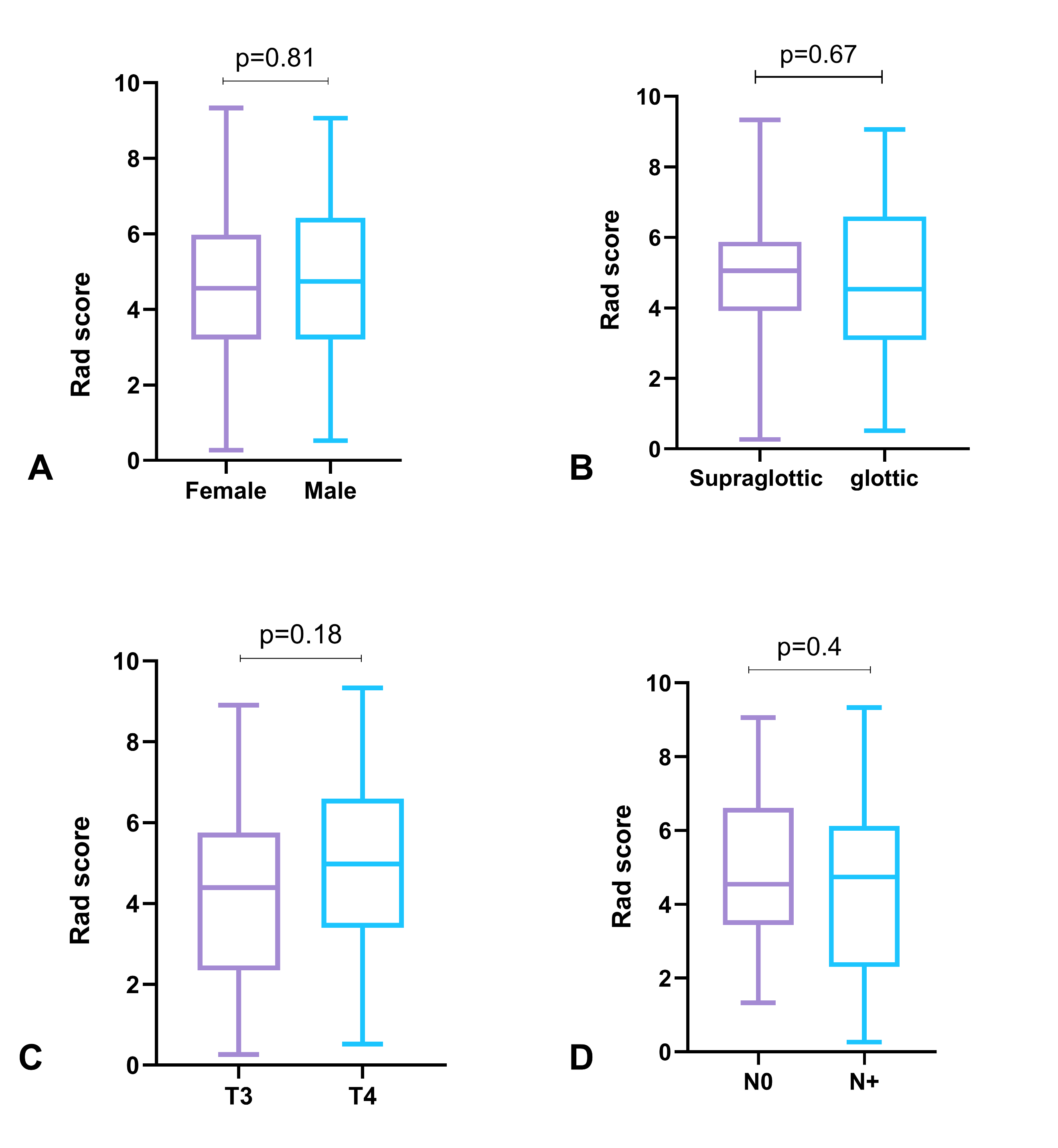
**

**Figure S3. Relationships between the Rad-score and** **clinical features. No significant correlation between radiomic score with gender(A), tumor site(B),T(C)，N(D).**

**Supplementary Table 4**

**Table 4 Univariate and multivariate analysis with Cox proportional hazard model in validation cohort.**

| **Covariates** | Univariate | |  | | multivariate | | | |  |
| --- | --- | --- | --- | --- | --- | --- | --- | --- | --- |
|  | **HR（95%CI）** | | ***P* value** | | | **HR（95%CI）** | | **Adjusted *P* value** | |
| **Rad-score** | 1.56(1.17-3.43) | | | 0.02 | | 1.78(1.08-2.95) | | 0.04 | |
| **N Stage** | 1.98(1.69-3.19) | | | 0.01 | | 2.03(1.51-3.09) | | 0.04 | |
| **Volume** | 1.26(1.05-1.44) | | | 0.002 | | 1.16(1.12-1.53) | | 0.015 | |
| **PR status** | 1.28(1.21-2.34) | | | 0.03 | | 1.43(1.15-3.48) | | 0.04 | |
| **T Stage** | 1.08(0.34-1.35) | | | 0.52 | | |  |  | |
| **Size** | 1.45(0.88-2.78) | | | 0.58 | | |  |  | |
| **Radiation** | 0.73(0.59-1.08) | | | 0.49 | | |  |  | |
| **Age** | 2.36(0.89-3.34) | | | 0.56 | | |  |  | |
| **Gender** | 0.72(0.21-1.46) | | | 0.73 | | |  |  | |
| **Site** | | 0.63(0.61-1.83) | | 0.15 | | |  |  | |

Multivariate analysis was applied using the Cox proportional hazards(*P*<0.05) model to identify independent predictors of survival that involved the univariate variables. After a series of multivariate analyses, covariates with a P value < 0.05 were used for subsequent model construction. HR: hazard ratios.
